# Perinatal Garlic Oil Supplementation Averts Rat Offspring Hypertension Programmed by Maternal Chronic Kidney Disease

**DOI:** 10.3390/nu14214624

**Published:** 2022-11-02

**Authors:** You-Lin Tain, Chih-Yao Hou, Guo-Ping Chang-Chien, Sufan Lin, Chien-Ning Hsu

**Affiliations:** 1 Department of Pediatrics, Kaohsiung Chang Gung Memorial Hospital, Kaohsiung 833, Taiwan; 2 College of Medicine, Chang Gung University, Taoyuan 330, Taiwan; 3 Department of Seafood Science, National Kaohsiung University of Science and Technology, Kaohsiung 811, Taiwan; 4 Institute of Environmental Toxin and Emerging-Contaminant, Cheng Shiu University, Kaohsiung 833, Taiwan; 5 Center for Environmental Toxin and Emerging-Contaminant Research, Cheng Shiu University, Kaohsiung 833, Taiwan; 6 Super Micro Mass Research and Technology Center, Cheng Shiu University, Kaohsiung 833, Taiwan; 7 Department of Pharmacy, Kaohsiung Chang Gung Memorial Hospital, Kaohsiung 833, Taiwan; 8 School of Pharmacy, Kaohsiung Medical University, Kaohsiung 807, Taiwan

**Keywords:** garlic, hypertension, hydrogen sulfide, developmental origins of health and disease (DOHaD), gut microbiota, renin-angiotensin system, nitric oxide

## Abstract

Garlic (*Allium sativum*) is a functional food, having hydrogen sulfide (H_2_S)-releasing capacity, which exhibits considerable effects on hypertension and gut microbiota. H_2_S is strongly associated with hypertension and chronic kidney disease (CKD). Maternal CKD leads to hypertension in adult rat progeny, which was linked to disruption of the gut microbiota. This study validated the benefits of perinatal garlic oil supplementation against offspring hypertension induced by maternal CKD via modulation of H_2_S signaling, nitric oxide (NO), and the gut microbiota. Before pregnancy, female rats received a 0.5% adenine diet for 3 weeks to develop an animal model to mimic human CKD. Garlic oil (100 mg/kg/day) or vehicle was administered to pregnant rats by oral gavage during gestation and lactation. Perinatal garlic oil supplementation protected against maternal CKD-induced hypertension in offspring at 12 weeks of age. The beneficial effects of garlic oil are associated with enhanced H_2_S signaling, increased NO bioavailability, and shifts in gut microbiota. Perinatal garlic oil supplementation reduces abundance of genera *Variovorax*, *Nocardia*, *Sphingomonas*, and *Rhodococcus*. Our findings provide insight into the role of early H_2_S-targeted intervention as a preventive strategy in hypertension for further translational research.

## 1. Introduction

Hydrogen sulfide (H_2_S) is a gaseous molecule with a biological impact on human health and disease [1,2]. Even though H_2_S is recognized as a toxic gas, it has an essential biofunctional role at the physiological level [2]. Importantly, H_2_S signaling participates in the regulation of renal physiology and blood pressure (BP) [1,3]. The formation of H_2_S can occur through the enzymatic pathway, non-enzymatic pathway, and gut microbial origins [1,4]. H_2_S is enzymatically synthesized from L-cysteine via three enzymes: 3-mercaptopyruvate sulfurtransferase (3MST), cystathionine γ-lyase (CSE), and cystathionine β-synthase (CBS) [1]. In addition, non-enzymatic synthesis of H_2_S can occur in organic thiol. Thiosulfate can be reduced and regenerate H_2_S [5]. Rather, thiosulfate is a major oxidation product formed during H_2_S metabolism. As a result, thiosulfate may reflect H_2_S recycling because it is not only a metabolite of H_2_S, but also an index of the sulfide pool [6]. Of note, half of fecal H_2_S is generated from gut microbiota [4]. The sulfate-reducing bacteria (SRB) represent a non-enzymatic source of fecal H_2_S. Additionally, gut bacteria can enzymatically produce H_2_S by sulfite reduction. Increasing evidence suggests the key role of H_2_S in hypertension and kidney disease [3,6,7]. Nevertheless, limited information is available on whether the dysregulated H_2_S signaling pathway is involved in the gut–kidney axis, leading to the development of kidney disease and hypertension.

Adult disease can originate from an adverse intrauterine environment, which is referred to as the developmental origins of health and disease (DOHaD) [8]. About 3–4% of childbearing-aged women have chronic kidney disease (CKD) [9]. Maternal CKD is closely connected to adverse maternal and offspring outcomes [10]. Recent evidence suggests the interactions between gut microbiota and the kidney through the gut–kidney axis contribute to hypertension [11]. We previously found that maternal adenine-induced CKD induced hypertension in adult rat offspring, which was accompanied by disruption of the gut microbiota [12]. Conversely, maternal CKD-primed offspring hypertension can be protected by maternal L-cysteine supplementation, suggesting the protective role of H_2_S [13].

Garlic (*Allium sativum*), a popular functional food, is rich in natural polysulfides as a dietary source of H_2_S donors [14], which confer a health benefit due to its antihypertensive effect [15]. The beneficial effects of H_2_S in hypertension are associated with reducing oxidative stress, increasing nitric oxide (NO) bioavailability, rebalancing of the renin-angiotensin aldosterone system (RAAS), and altering the gut microbiota [1,2,3,16]. Our prior work indicates that maternal garlic oil supplementation protects hypertension in offspring born to high-fat diet-fed dams, and its protective effects are associated with enhancing the H_2_S signaling system and shaping gut microbiota [17]. The aim of this study was to assess whether perinatal garlic oil supplementation has the potential to avert maternal CKD-induced offspring hypertension and whether its protective effects are associated with enhancement of H_2_S signaling, increases in NO bioavailability, and shifts in gut microbiota composition. 

## 2. Materials and Methods

### 2.1. Animal Care and Experimental Design

The procedures carried out on the animals were approved by our Institutional Animal Ethics Committee (Permit # 2020110202) and according to the rules of the Care and Use of Laboratory Animals of the National Institutes of Health. We purchased virgin Sprague Dawley (SD) rats from BioLASCO Taiwan Co. Ltd. (Taipei, Taiwan) for breeding. Rats were housed in our AAALAC-accredited animal facility.

To conduct a CKD model, eight-week-old female rats were fed with chow containing 0.5% adenine for 3 weeks before pregnancy [12]. Female rats were successfully mated with a male rat at 11 weeks of age, as confirmed by the occurrence of a copulatory plug. We randomly divided the dams into four groups: CN (control), CKD (adenine-treated rats), CN+GO (control rats received garlic oil), and CKD+GO (adenine-treated rats received garlic oil). Daily administration of garlic oil (GO, Sigma-Aldrich, St. Louis, MO, USA) or vehicle by oral gavage at the dose of 100 mg/kg/day was carried out during gestation and lactation according to prior work in rats [16]. All litters were standardized to eight pups at one day of age. As males have a higher likelihood of hypertension and have hypertension earlier than females [18], we only included male progeny from each litter for the experiment. 

BP was measured every four weeks using the CODA rat tail-cuff system (Kent Scientific Corporation, Torrington, CT, USA). One week prior to the actual recording sessions, rat offspring were allowed to adapt to the restraint chamber. A total of 32 rats belonging to four experimental groups (n = 8 per group) were sacrificed at 12 weeks old. Prior to sacrifice, fecal samples were collected in the morning and kept at −80 °C until analyses. The renal cortex and medulla were separated and subsequently snap-frozen at −80 °C. Blood samples were collected using heparin tubes.

### 2.2. Analysis of NO Parameters by HPLC

As L-arginine is the substrate for NO synthase (NOS), and symmetric and asymmetric dimethylarginine (SDMA and ADMA) are NOS inhibitors, their levels were determined using HPLC (HP series 1100, Agilent Technologies, Inc., Santa Clara, CA, USA) with the OPA-3MPA derivatization reagent based on our previously described method [12].

### 2.3. Analysis of Plasma H_2_S and Thiosulfate Levels by HPLC-MS

Plasma H_2_S and thiosulfate concentrations were measured by an HPLC-Mass Spectrometry (MS) protocol previously validated in our lab [19] using an Agilent Technologies 1290 HPLC system together with an Agilent 6470 Triple Quadrupole LC/MS (Agilent Technologies, Wilmington, DE, USA) and an electrospray ionization (ESI) source [18]. Chromatographic separation was carried out using a Supelco C18 column (3 µm, 50 × 2.1 mm; Sigma–Aldrich) protected by an Ascentis C18 column (3 µm, 20 × 2.1 mm, Merck KGaA, Darmstadt, Germany). Solvents used in the elution step were composed of 0.1% formic acid (v/v) in acetonitrile, at a flow rate of 300 µL/min. We measured thiosulfate derivative pentafluorobenzyl (PFB)-S_2_O_3_H and H_2_S derivative sulfide dibimane (SDB). Selected reaction monitoring mode was utilized to detect target compounds with a targeted 415→223 *m/z* and 292.99→81 *m/z*, for SDB and PFB-S_2_O_3_H, respectively. Phenyl 4-hydroxybenzoate (PHB) was used as an internal standard and detection was at 212.99→93 *m/z*. The percentage of coefficient of variation for the intra-assay variability was 4% and 6% for H_2_S and thiosulfate, respectively.

### 2.4. Analysis of RAAS Components by qPCR

RNA was extracted from the renal cortical tissues. RAAS components’ mRNA levels were analyzed by quantitative polymerase chain reaction (qPCR) in duplicate using SYBR Green; results were normalized to the 18S ribosomal RNA (R18S), as previously described [12,13]. The primers for renin, angiotensinogen (AGT), angiotensin converting enzyme-1 (ACE1) and -2 (ACE2), angiotensin II type 1 receptor (AT1R), and angiotensin-(1–7)/Mas receptor (MAS) are provided in Table 1. The relative mRNA expression levels of genes were calculated using the comparative CT method. The fold change for gene expression relative to the control was calculated using the formula 2^−ΔΔCT^.

### 2.5. Analysis of H_2_S-Producing Enzymes by Western Blot

As stated previously [19], renal cortex homogenates were prepared for Western blotting with antibody incubation. A total of 200 µg protein was loaded and electrophoresed through a 10% polyacrylamide gel. Electrophoretically separated proteins were electrotransferred onto a nitrocellulose membrane (GE Healthcare Bio-Sciences Corp., Piscataway, NJ, USA). In this procedure, Ponceau S staining (PonS, Sigma-Aldrich) was utilized to correct for variations in the total protein loading. For the detection of H_2_S-producing enzymes, the membranes were incubated with a mouse monoclonal antibody against rat CBS (1:1000, overnight incubation; Abnova Corporation, Taipei, Taiwan), a rabbit monoclonal antibody against rat 3MST (1:500, overnight incubation; Novus Biologicals, Littleton, CO, USA), or a rabbit polyclonal antibody against rat CSE (1:1000, overnight incubation; Proteintech Group, Inc. Chicago, IL, USA). The protein abundance of the samples was quantified using Quantity One Analysis software (Bio-Rad) following enhanced chemiluminescence reagent detection (PerkinElmer, Waltham, MA, USA). Band density was represented as the integrated optical density (IOD)/PonS to correct the variations in total protein loading. 

### 2.6. Metagenomics Analysis of Gut Microbiota

Fecal genomic DNA was extracted and analyzed by 16S rRNA gene-based metagenomics analysis at Biotools Co., Ltd. (New Taipei City, Taiwan), as we described previously [20]. The full-length 16S genes were amplified with barcoded multiplexed primers for SMRTbell library preparation and sequencing (PacBio, Menlo Park, CA, USA). The QIIME2 was utilized to process data from a high-throughput 16S rRNA sequencing. A phylogenetic tree was derived from the amplicon sequence variants (ASVs) via FastTree (QIIME2) [21]. We computed two α-diversity metrics to measure the richness and evenness of the communities by Faith’s phylogenetic diversity (PD) index and the Shannon index. The similarities between communities across groups (i.e. β-diversity) were examined using the analysis of similarities (ANOSIM) and principal coordinate analysis (PCoA) based on the unweighted UniFrac distance. 

### 2.7. Statistics

All data are presented as means ± the standard error of the mean (SEM), and *p* < 0.05 was considered statistically significant. Statistical analyses were carried out by one-way ANOVA or two-way ANOVA where appropriate. To produce post hoc multiple comparison tests, Tukey’s post hoc test was utilized. Statistical analysis was performed using SPSS (SPSS Inc., Chicago, IL, USA).

## 3. Results

### 3.1. Offspring Blood Pressure and Weight

**Figure 1:**
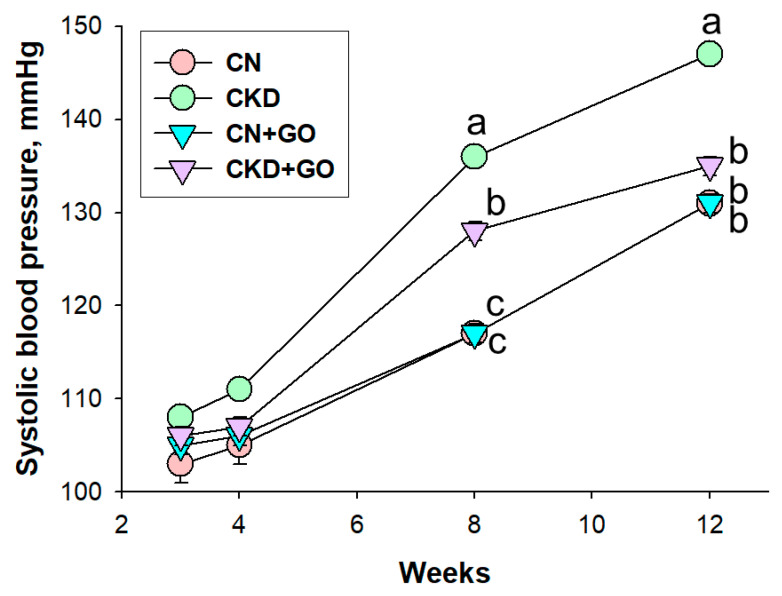
Effect of perinatal garlic oil supplementation on systolic blood pressure in offspring at 3–12 weeks of age. The letters a, b and c indicate the differences between the groups (*p* < 0.05, two-way ANOVA); N = 8/group.

Table 2 shows the pup mortality rate was zero. The body weight (BW) was not affected by maternal CKD or perinatal garlic oil supplementation. The kidney weight (KW) and the KW-to-BW ratio were not different among the four groups. However, at 12 weeks of age, male offspring of dams with CKD had higher systolic and diastolic BPs, and mean arterial pressure, than male offspring of dams without CKD. Conversely, perinatal garlic oil supplementation averted the elevation of BP. Figure 1 illustrates that longitudinal measurement of BP between week 3 and week 12. Similarly, systolic BP elevations started at week 8 in CKD offspring, which was averted by garlic oil treatment. Overall, these findings indicate that maternal CKD induced offspring hypertension, which was averted by perinatal garlic oil supplementation. 

### 3.2. H_2_S Pathway

**Figure 2:**
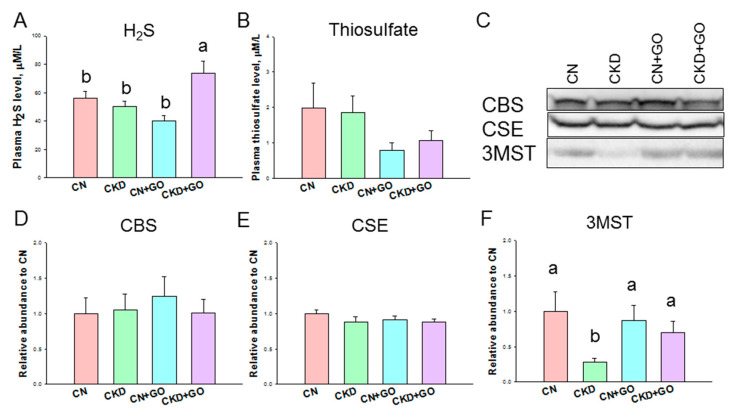
Effect of perinatal garlic oil supplementation on (**A**) plasma level of H_2_S and (**B**) thiosulfate, and H_2_S-producing enzymes in 12-week-old offspring kidneys. (**C**) Representative Western blots demonstrate cystathionine β-synthase (CBS, ~61 kDa), cystathionine γ-lyase (CSE, ~45 kDa), and 3-mercaptopyruvate sulfurtransferase (3MST, ~52 kDa) bands. The relative protein levels of renal cortical (**D**) CBS, (**E**) CSE, and (**F**) 3MST were calculated. The letters a and b indicate the differences between the groups (*p* < 0.005, one-way ANOVA); N = 8/group.

Figure 2 illustrates the H_2_S pathway, which includes plasma H_2_S and thiosulfate levels and protein levels of H_2_S-producing enzymes in offspring kidneys. The plasma H_2_S level was higher in the CKD+GO group compared to the three other groups (Figure 2A), whereas the plasma thiosulfate level was comparable among the four groups (Figure 2B). Renal protein abundance of H_2_S-producing enzymes CBS, CSE, and 3MST is compared in Figure 2C. Garlic oil supplementation has a negligible effect on renal CBE and CSE abundance (Figure 2D,E). Maternal CKD led to a reduction in the renal 3MST protein level, which garlic oil supplementation prevented (Figure 2F). Together, these findings show that garlic oil treatment increases plasma H_2_S and the renal 3MST protein level.

### 3.3. NO Pathway

**Figure 3:**
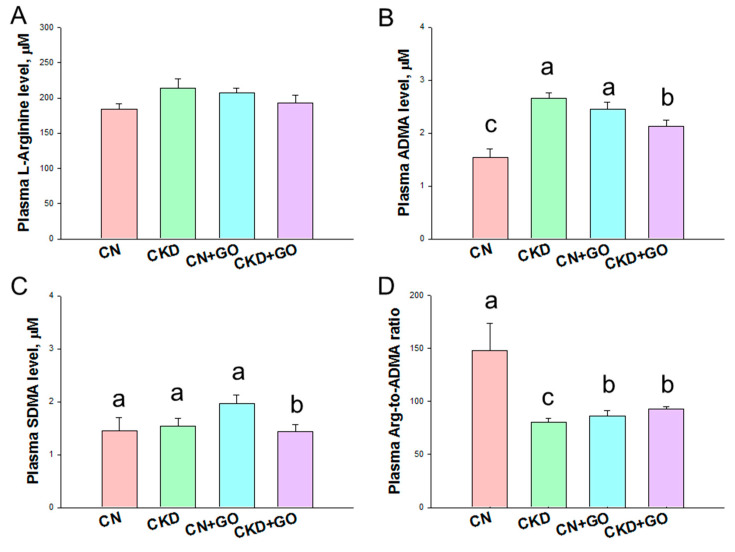
Effect of perinatal garlic oil supplementation on NO pathway in 12-week-old offspring. These NO parameters include (**A**) L-arginine, (**B**) asymmetric dimethylarginine (ADMA), (**C**) symmetric dimethylarginine (SDMA), and (**D**) the ratio of L-arginine-to-ADMA. The letters a, b and c indicate the differences between the groups (*p* < 0.05, one-way ANOVA); N = 8/group.

Plasma NO-related parameters are compared in Figure 3. The plasma level of L-arginine, the substrate for NOS to generate NO, was not different among the four groups (Figure 3A). Maternal CKD caused an increase in the plasma ADMA level, which was prevented by perinatal garlic oil supplementation (Figure 3B).

Compared to the CN+GO group, the plasma SDMA level was lower in the CKD+GO group (Figure 3C). Additionally, CKD reduced the L-arginine-to-ADMA ratio, an index of NO bioavailability [22], in the CKD group (Figure 3D). In contrast, the decreased L-arginine-to-ADMA ratio was improved by garlic oil supplementation. 

### 3.4. The RAAS

**Figure 4:**
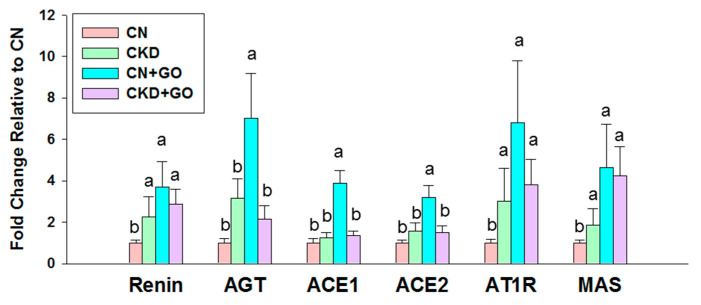
Effect of perinatal garlic oil supplementation on the renin-angiotensin aldosterone system (RAAS) in offspring kidneys at 12 weeks of age. The letters a and b indicate the differences between the groups (*p* < 0.05, one-way ANOVA); N = 8/group.

As aberrant activation of the RAAS participates in hypertension of developmental origins [23], we then looked at the renal mRNA expression of RAAS components. Compared to the CN group, renal mRNA expression of renin, AGT, ACE1, ACE2, AT1R, and MAS was higher in the CN+GO group (Figure 4). That is, two axes of RAAS—classical axis (ACE1/Angiotensin II) and nonclassical axis (ACE2/Ang-(1–7))—are both activated by perinatal garlic oil treatment. Additionally, garlic oil supplementation enhanced AT1R and MAS expression in the offspring kidneys of the CKD+GO group vs. the CKD group. It is known that activating AT1R promotes vasoconstriction, while agonizing MAS in favor of vasodilatation. As the two RAAS axes act in an opposite manner, our data suggest that the RAAS may not be the primary mechanism behind the protective effect of garlic oil on maternal CKD-induced hypertension.

### 3.5. Alterations in Gut Microbiota

**Figure 5:**
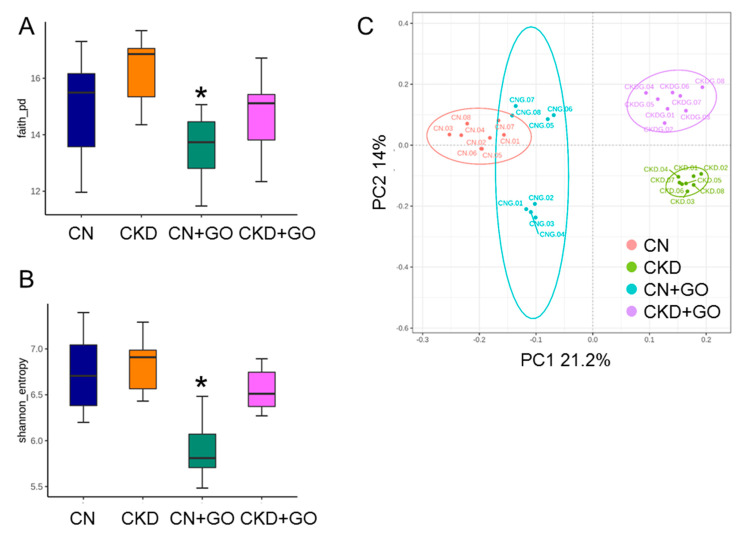
Comparison of α-diversity among four groups of 12-week-old offspring in (**A**) Faith’s phylogenetic diversity (PD) index and (**B**) Shannon index; (**C**) principal coordinate analysis (PCoA) plots of β-diversity calculated by the unweighted UniFrac metric across the four groups; each point represents the microbiota of a single sample, and colors reflect metadata for that sample. * *p* < 0.05 vs. CN.

We first analyzed α- and β-diversity metrics to elucidate how maternal CKD and perinatal garlic oil supplementation influence the establishment of gut microbiota in adult offspring. Community richness and evenness were estimated by Faith’s PD index and the Shannon index, respectively. We found maternal CKD has a negligible effect on both α-diversity metrics, while these metrics were lower in the CN+GO group than those in the CN group (Figure 5A,B). PCoA plots of β-diversity based on the unweighted UniFrac metric were utilized to illuminate the samples clustered according to study groups, as shown in Figure 5C. We observed from ANOSIM that there were significant differences in the groups (all *p* < 0.05). Our data reveal that perinatal garlic oil supplementation caused offspring gut microbiota shifts in diversity. Regarding the composition, the predominant phyla are *Firmicutes* and *Bacteroidetes*, followed by *Actinobacteria*, *Deferribacteres*, and *Proteobacteria*; these results are consistent with prior animal studies that showed gut microbiota are dominated by these bacteria [11,12,13].

**Figure 6:**
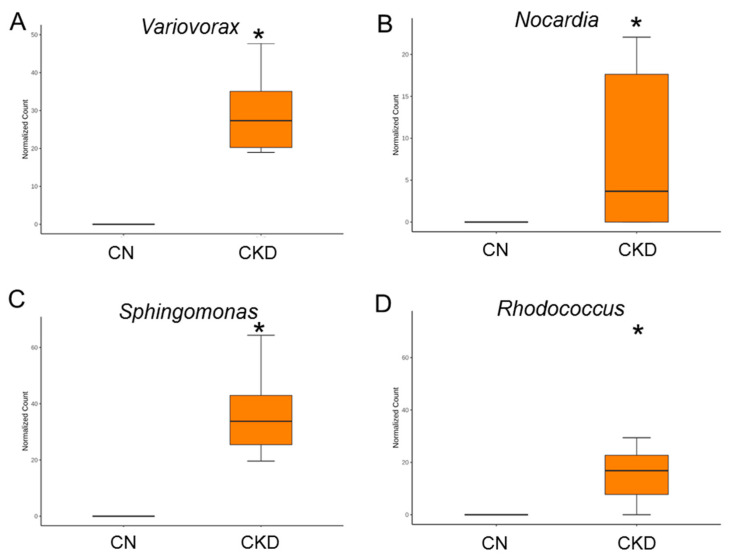
Effect of maternal CKD on the gut microbiota in 12-week-old male offspring. Genus-level relative abundance of (**A**) *Variovorax*, (**B**) *Nocardia*, (**C**) *Sphingomonas*, and (**D**) *Rhodococcus*. * *p* < 0.05.

**Figure 7:**
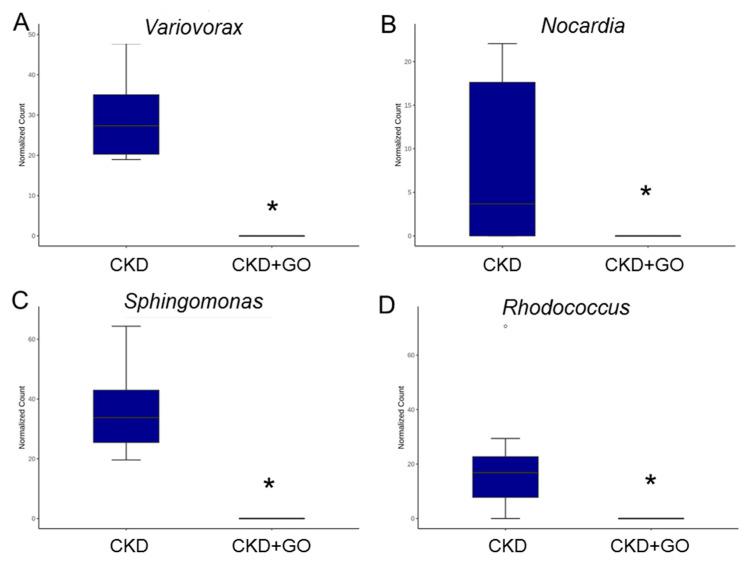
Effect of perinatal garlic oil on the gut microbiota of CKD+GO group compared to the CKD group. Genus-level relative abundance of (**A**) *Variovorax*, (**B**) *Nocardia*, (**C**) *Sphingomonas*, and (**D**) *Rhodococcus*. * *p* < 0.05.

Maternal CKD caused a greater proportion of genera *Variovorax Nocardia, Sphingomonas*, and *Rhodococcus* in the CKD group vs. the CN group (Figure 6A–D). On the contrary, the proportion of these enriched genera was lowered by perinatal garlic acid supplementation (Figure 7A–D). 

## 4. Discussion

Our results provide novel insights into the protective roles of perinatal garlic oil supplementation on maternal CKD-induced offspring hypertension through regulation of the H_2_S signaling, shifts in gut microbiota, and restoration of NO. The major findings are described as follows: (1) perinatal supplementation with garlic oil prevented the increase in BP in male offspring born to dams with CKD at 12 weeks of age; (2) perinatal garlic oil supplementation increased renal 3MST protein levels and the plasma H_2_S level; (3) the benefits of garlic oil for offspring hypertension were linked to increases in NO bioavailability, characterized by decreases in ADMA and increases in the L-arginine-to-ADMA ratio; (4) perinatal garlic oil supplementation led to gut microbiota shifts in diversity and composition; and (5) the advantageous effect of garlic oil against offspring hypertension coincided with decreases in the genera *Variovorax*, *Nocardia*, *Sphingomonas*, and *Rhodococcus*.

Although prior research suggests the BP-lowering effect of garlic [15,17], our report goes beyond previous reports and demonstrates that perinatal garlic oil supplementation averts maternal CKD-induced offspring hypertension. Considering sulfur-containing compounds derived from garlic are natural precursors of H_2_S [14], the findings of the current research agree with past findings, suggesting that H_2_S plays a role in the development of hypertension [7,24].

The protective effect of garlic oil against maternal CKD-induced hypertension may be relevant to its ability to increase renal 3MST protein levels and the plasma H_2_S level. The findings of this study are consistent with prior research, which showed that the uses of different H_2_S-based interventions in early life to increase H_2_S bioavailability may be a reprogramming approach to avert offspring hypertension in several models of developmental origin [17,19,25].

Another protective mechanism of garlic oil may also be associated with increased NO bioavailability. Increasing evidence has indicated that early-life interventions targeting the NO pathway can be a preventive strategy to avert the development of hypertension [26]. This concept is corroborated by our present study, which showed that the beneficial effects of garlic oil coincided with increased NO bioavailability, represented by a decrease in ADMA and an increase in the ratio of L-arginine-to-ADMA. A previous study revealed sodium hydrosulfide, a H_2_S donor, can rescue NO bioavailability and prevent hypertension in a NO deficiency rat model [27]. As such, there may be a crosstalk between H_2_S and NO in the control of BP. Accordingly, maternal CKD-induced hypertension may be counterbalanced by the garlic oil-mediating NO signaling pathway and shifted toward vasodilation. 

Our findings support results of previous human studies showing the pathophysiological importance of H_2_S and NO bioavailability during cardiovascular disease [28,29]. Although H_2_S and its metabolites have been utilized as biomarkers for several human diseases [30], different analytical methods have obvious limitations and provide contradictory results, especially in the clinical setting [28,29,30,31]. A previous study reported that elevation of plasma-free H_2_S found in patients with peripheral arterial disease was due to a compensatory response to endothelial dysfunction and dysregulation of NO bioavailability [28]. Using the animal model, we not only detected plasma H_2_S and thiosulfate levels, but also tissue H_2_S-producing enzymes. Our data suggest that protection by garlic oil supplementation against maternal CKD-primed offspring hypertension can be attributed to increased renal 3MST protein levels, the plasma H_2_S level, and NO bioavailability. Thus, future work in developing an ideal methodology to reduce the gap between human and animal research is required to better assess H_2_S detection in clinical practice. 

Additionally, the beneficial effect of garlic oil is possibly due to alterations of the gut microbiota. Consistent with findings in hypertensive humans and animals [32,33,34], a high abundance of the genera *Variovorax*, *Sphingomonas*, and *Rhodococcus* was identified as a microbial marker for hypertension in the maternal CKD-induced hypertension model. Although *Nocardia* spp. are opportunistic bacteria related to kidney disease and hypertension in humans [35], the association between high BP and a high *Nocardia* abundance was addressed, for the first time, by our study. 

Since the gut is another major source of H_2_S [4], we also determined the composition of gut microbiota with a focus on sulfate- or sulfite-reducing bacteria. We observed almost all SRB (e.g., *Desulfobacter* and *Desulfovibrio*) were unnoticeable in both groups receiving garlic oil. Sulfite reductase exists in several species, such as *E coli, Salmonella, Klebsiella, Corynebacterium, Rhodococcus,* and *Baccilus* [36]. Our current data showed the abundance of most sulfite-reducing bacteria was unaltered in response to perinatal garlic oil supplementation, with the exception that the abundance of genus *Rhodococcus* was reduced by garlic oil. Hence, whether the protective role of garlic oil is related to gut microbiota-derived H_2_S and alterations of sulfate- or sulfite-reducing bacteria awaits further clarification. Surprisingly, we observed that perinatal garlic oil supplementation reduced indices of richness and evenness in offspring gut microbiota. As the decrease in α-diversity has been linked to disease risk [37], further studies of the long-term outcomes or moderating role of garlic oil in normal controls is therefore needed.

In addition to H_2_S and the NO system, accumulated evidence suggests that early blockade of the RAAS affords protection against the offspring hypertension programmed by various maternal insults [23]. However, our data suggested that the RAAS is less likely to be a primary protective mechanism of garlic oil against maternal CKD-programmed hypertension as RAAS components were not different between the CKD and CKD+GO groups. 

This study has some limitations. One limitation is that gut microbiota analysis was only carried out in adult offspring, but not in mothers and young progeny. Whether perinatal garlic oil supplementation can regulate gut microbes involved in H_2_S metabolism in both dams and neonate offspring, and whether gut microbiota-derived fecal H_2_S is associated with offspring outcomes later in life, both deserve further evaluation. Secondly, we only investigated male offspring. Further work is required to distinguish whether garlic oil has a sex-specific effect and whether sex differences exist behind maternal CKD-induced programmed hypertension. Finally, in addition to organosulfur, garlic contains important biological compounds such as polyphenols [38]. Given that the major active compounds of garlic oil were not determined, the extent of its protective effect throughout the H_2_S signaling pathway deserves additional research.

## 5. Conclusions

In conclusion, our study demonstrated that perinatal garlic oil supplementation averted offspring hypertension programmed by maternal CKD. There are several protective mechanisms by which garlic oil therapy protects adult offspring against hypertension, including enhancement of the renal H_2_S-generating system, increases in NO bioavailability, and shifts in gut microbiota. Our pre-clinical investigation provides an in-depth understanding of the perinatal use of functional foods targeting H_2_S signaling and impacting offspring hypertension. This may help us develop effective reprogramming strategies to avert hypertension in children born to mothers with CKD.

## Figures and Tables

**Table 1:** PCR primer sequences.

Gene	Forward	Reverse
Renin	5 aacattaccagggcaactttcact 3	5 acccccttcatggtgatctg 3
AGT	5 gcccaggtcgcgatgat 3	5 tgtacaagatgctgagtgaggcaa 3
ACE1	5 caccggcaaggtctgctt 3	5 cttggcatagtttcgtgaggaa 3
ACE2	5 acccttcttacatcagccctactg 3	5 tgtccaaaacctaccccacatat 3
AT1R	5 gctgggcaacgagtttgtct 3	5 cagtccttcagctggatcttca 3
MAS	5 catctctcctctcggctttgtg 3	5 cctcatccggaagcaaagg 3
R18S	5 gccgcggtaattccagctcca 3	5 cccgcccgctcccaagatc 3

AGT = angiotensinogen; ACE = angiotensin converting enzyme; ACE2 = angiotensin converting enzyme-2; AT1R = angiotensin II type 1 receptor; MAS = angiotensin-(1–7)/Mas receptor, R18S = 18S ribosomal RNA.

**Table 2:** Weights and blood pressure.

Groups	CN	CKD	CN+GO	CKD+GO
Mortality	0%	0%	0%	0%
Body weight (BW), g	277 ± 14	286 ± 11	285 ± 12	287 ± 11
Left kidney weight (KW), g	1.3 ± 0.06	1.29 ± 0.04	1.39 ± 0.110	1.27 ± 0.062
Left KW/100 g BW	0.47 ± 0.01	0.45 ± 0.01	0.49 ± 0.029	0.44 ± 0.015
Systolic blood pressure, mmHg	131 ±1 ^b^	147 ± 1 ^a^	131 ±1 ^b^	135 ±1 ^b^
Diastolic blood pressure, mmHg	87 ± 1 ^b^	101 ± 2 ^a^	87 ± 2 ^b^	87 ± 3 ^b^
Mean arterial pressure, mmHg	102 ± 1 ^b^	117 ± 1 ^a^	102 ± 1 ^b^	103 ± 2 ^b^

N = 8/group; the letters ^a^ and ^b^ indicate the differences between the groups (*p* < 0.05, one-way ANOVA).

## Data Availability

Data are contained within the article.
